# Treatment of Diarrhea and Dysentery of Fever

**Published:** 1850-03

**Authors:** 


*Treatment of the Diarrhea and Dysentery of Fever.*—Dr. Hudson speaks highly of turpentine, in doses of six or eight minims, given in emulsion, in the treatment of this affection. He furnishes corroborative testimony of its value.

